# Supplementary figures and images for: Energy and Key Micronutrient Intake in Amateur Swimmers: A Pilot Study
Source: Nutrients. 2025 Feb 13;17(4):664. doi: 10.3390/nu17040664 (PMC11858584; doi:10.3390/nu17040664)

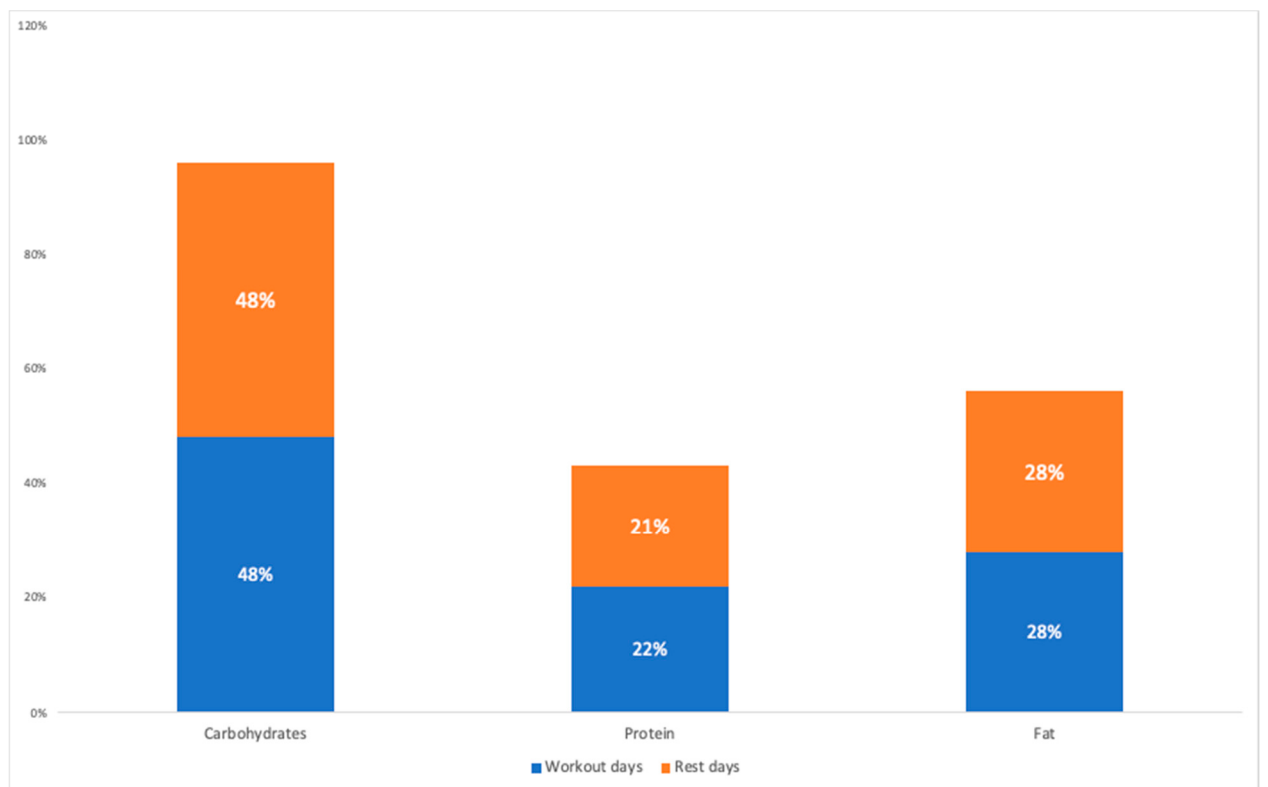

Figure S1 – Macronutrient contribution to energy intake in Workout days and Rest days.

Supplement: Supplementary file 1 [file nutrients-17-00664-s001.zip › nutrients-3456716-supplementary.pdf]
